# Point mutation of the xylose reductase (XR) gene reduces xylitol accumulation and increases citric acid production in *Aspergillus carbonarius*

**DOI:** 10.1007/s10295-014-1415-6

**Published:** 2014-02-26

**Authors:** István Weyda, Mette Lübeck, Birgitte K. Ahring, Peter S. Lübeck

**Affiliations:** 1 Section for Sustainable Biotechnology, Aalborg University Copenhagen, A. C. Meyers Vænge 15, 2450 Copenhagen SV, Denmark; 2 Bioproducts, Sciences and Engineering Laboratory (BSEL), Washington State University Tri-Cities, 2710 Crimson Way, Richland, WA 99354 USA

**Keywords:** Xylose fermentation, Xylitol, *Aspergilluscarbonarius*, Pentose catabolic pathway, Xylose reductase, Citric acid

## Abstract

*Aspergillus carbonarius* accumulates xylitol when it grows on d-xylose. In fungi, d-xylose is reduced to xylitol by the NAD(P)H-dependent xylose reductase (XR). Xylitol is then further oxidized by the NAD^+^-dependent xylitol dehydrogenase (XDH). The cofactor impairment between the XR and XDH can lead to the accumulation of xylitol under oxygen-limiting conditions. Most of the XRs are NADPH dependent and contain a conserved Ile-Pro-Lys-Ser motif. The only known naturally occurring NADH-dependent XR (from *Candida parapsilosis*) carries an arginine residue instead of the lysine in this motif. In order to overcome xylitol accumulation in *A. carbonarius* a Lys-274 to Arg point mutation was introduced into the XR with the aim of changing the specificity toward NADH. The effect of the genetic engineering was examined in fermentation for citric acid production and xylitol accumulation by using d-xylose as the sole carbon source. Fermentation with the mutant strain showed a 2.8-fold reduction in xylitol accumulation and 4.5-fold increase in citric acid production compared to the wild-type strain. The fact that the mutant strain shows decreased xylitol levels is assumed to be associated with the capability of the mutated XR to use the NADH generated by the XDH, thus preventing the inhibition of XDH by the high levels of NADH and ensuring the flux of xylose through the pathway. This work shows that enhanced production of citric acid can be achieved using xylose as the sole carbon source by reducing accumulation of other by-products, such as xylitol.

## Introduction

Pentose sugars are among the major components of plant-based lignocellulosic biomass [35]. Future biorefineries, which employ plant materials as feedstock, require capable microbial strains for the cost-effective conversion of all the sugars of the biomass, including the pentose fraction, into final products such as advanced biofuels, bulk biochemicals, and high-value compounds.

*Aspergillus carbonarius*, a fungus closely related to *Aspergillus niger* [1], is able to consume a large variety of carbohydrates, including the two most abundant pentose sugars found in lignocellulosic biomass, d-xylose and l-arabinose ([42], unpublished data). Similar to *A. niger* [29], *A. carbonarius* naturally produces citric acid and acidifies its environment.

**Fig. 1:**
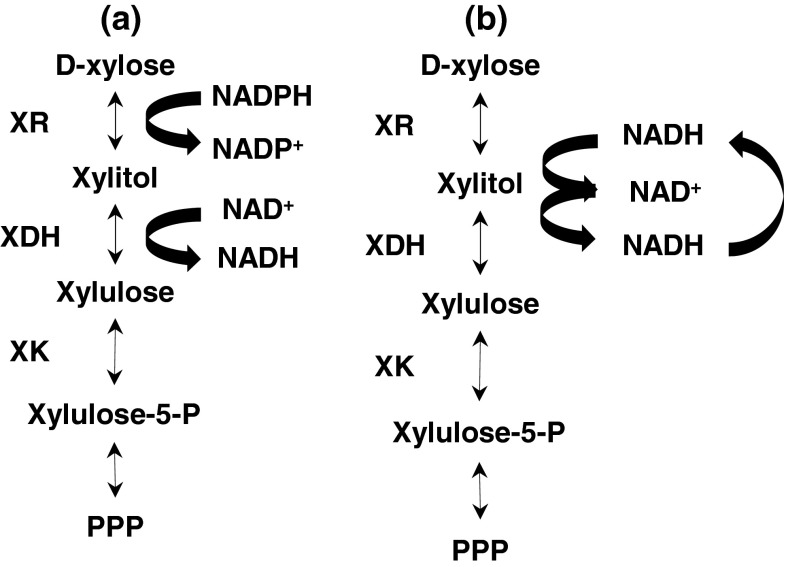
d-Xylose conversion in the PCP of **a** wild-type *A. carbonarius*, **b** mutant strain. *XR* xylose reductase, *XDH* xylitol dehydrogenase, *XK* xylulokinase, *PPP* pentose phosphate pathway

In eukaryotes, the conversion of d-xylose and l-arabinose occurs via the pentose catabolic pathway (PCP) [18]. This pathway involves a series of reversible reduction–oxidation steps followed by a phosphorylation step resulting in xylulose-5-phosphate, which is then channeled into the glycolysis via the pentose phosphate pathway (PPP) as described for *A. niger* [41] (Fig. 1a). The NAD(P)H-dependent d-xylose reductase (E.C. 1.1.1.307) is the first enzyme in the pathway, and catalyzes the reduction of d-xylose into the pathway intermediate, xylitol. *A. niger* harbors a xylose reductase (XR) belonging to aldo–keto reductase family 2 [16]. The majority of XRs belonging to this family are dependent on NADPH as the cofactor [24], some have dual specificity to both NADPH and NADH (e.g., *Candida tenuis*) [19]. However the consecutive enzyme of the PCP, xylitol dehydrogenase (XDH) (E.C. 1.1.1.9), requires NAD^+^ as the cofactor to carry out the electron transfer for the oxidation of xylitol into d-xylulose [2].The cofactor impairment between XR and XDH may lead to the accumulation of xylitol when the cells are under oxygen-limiting conditions where the availability of NAD^+^ becomes the limiting factor [9], thus d-xylose will not be further converted in the PCP.

Leitgeb et al. [24] described the effect of a single point mutation in the conserved Ile-Pro-Lys-Ser motif [22, 30] of the NADPH/NADH-dependent XR in *C. tenuis*. This point mutation consists of the replacement of Lys-274 with an Arg residue and results in conformational changes in the coenzyme binding pocket of the XR, thus altering the enzyme’s cofactor preference from NADPH towards NADH. This residue exchange was based on the fact that the only naturally occurring XR, from *Candida parapsilosis*, known to prefer NADH as the cofactor carries an arginine residue instead of the Lys-274 [23].

Several attempts were made in yeast to introduce the eukaryotic PCP or to alter existing pathway enzymes [3, 4, 13]. In most cases the accumulation of xylitol was observed, a direct result of the redox impairment between the XR and the XDH. In contrast to the eukaryotic pathway, the bacterial PCP does not include the two-step conversion of d-xylose to d-xylulose by the cofactor-dependent XR and XDH. Instead, this conversion is catalyzed by the xylose isomerase (XI) in a single-step reaction. Since the XI is not cofactor dependent, the bacterial pathway is redox neutral [18]. Only a few fungi and yeasts follow the isomerase-based PCP [15, 37]. Successful expression of bacterial XIs [5, 12], as well as eukaryotic XIs from *Pyromices* sp. [20] and *Orpinomyces* sp. [25], in *Saccharomyces cerevisiae* has resulted in high interest for engineering efficient xylose-fermenting strains for ethanol production.

*A. carbonarius* ITEM 5010 accumulates xylitol in citric acid fermentations with d-xylose as the sole carbon source. On the basis of the work of Leitgeb et al. [24], we hypothesized that the same Lys/Arg exchange in the XR gene in *A. carbonarius* would result in less xylitol accumulation and lead to an increased glycolytic flux. On the basis of the fact that the XR gene of *A. niger* had been previously identified [16] and owing to the close relation between the two *Aspergilli* we were able to identify a putative gene with high similarity to the *A. niger* XR gene and used that gene for site-directed mutagenesis. The effect of the mutation was studied in batch flask fermentation with d-xylose as the sole carbon source. Figure 1b illustrates the assumed conversion of d-xylose by the mutant strain after the alteration of cofactor specificity of the XR.

## Materials and methods

### Fungal strain and growth conditions

*Aspergillus carbonarius* ITEM 5010 wild-type strain, originally isolated from grapes in Italy [17], was used in this study for the site-directed mutagenesis of the XR gene and fermentation experiments. The mutant strain with the point mutation in the XR gene is indicated as SXR1 and was generated from the wild-type strain.

The strains were grown on Potato Dextrose Agar (Sigma) for 5–7 days at 30 °C for preparation of conidial suspensions. Sterile double distilled H_2_O (3 ml) was added to the plates to collect conidia from the surface of the agar, and the suspension was filtered through sterile Miracloth (Calbiochem). The strains were grown on minimal medium [34] supplemented with hygromycin B (100 μg ml^−1^) for selection of transformants.

### Site-directed mutagenesis and construction of USER-compatible fragments

The XR gene was identified in the full genome sequence of *A. carbonarius* [11] on the basis of sequence homology to the XR gene of *A. niger* (accession number AF219625). The XR gene was amplified together with 1-kb upstream and 1-kb downstream regions from genomic DNA of *A. carbonarius* by using Phusion High-Fidelity DNA polymerase (New England Biolabs) and oligonucleotides IW17 and IW24 (Table 1) according to manufacturer’s recommendation. The PCR resulted in a 3-kb-long fragment, which was further used as the template in the consecutive PCRs involving the mutation of the XR gene and amplification of up- and downstream homologous fragments. The resultant PCR fragment was inserted in the USER cloning vector pSB411 using the USER cloning technique as described by Nour-Eldin et al. [28] and modified by Hansen et al. [14].

**Table 1:** Oligonucleotide primers used in this study

Oligonucleotide	5′–3′ sequence
IW2	GCCACAGCTACGAGTTCATG
IW7	CATATGGCTAGACTTATCGACG
IW17	GGCACATTCCAACCATAGTCCA
IW24	ACCCGCTCAGGCACATGCTT
IW28	GGGTTTAAUCGTCCTGGATGCCAACTACC
IW29	GGACTTAAUTGTGATTGGGAGTGAGGTCTGA
IW30	AGAGCGAUATGGCCTCCCCTACCGTCA
IW31	TCTGCGAUCTAGAAGATGGGAGCGTAGAGGC
IW32	GGCATTAAUACAATCTGACTCCTATAAATCTC
IW33	GGTCTTAAUGCCAAATAATCCAACGGAA
IW40	GCAGTTATTCCCCGTAGTAATAACC
IW41	GGTTATTACTACGGGGAATAACTGCA
IW42	CGTTCGTGCTCGTACTCCTGTC
IW43	GTCGTGCCCCTCTTTAACCTCC
IW50	TCAGCGAGAGCCTGACCTAT
IW51	GATGTTGGCGACCTCGTATT

The point mutation of Lys-274 to Arg was carried out using the overlap extension method described elsewhere [33] using oligonucleotides IW40, IW41, IW42, and IW43 (Table 1) and verified by DNA sequencing using the sequencing service from Starseq (http://www.starseq.com). A fragment obtained by this method contained the mutated XR gene, which was made compatible for simple USER cloning with uracil-containing oligonucleotides IW30 and IW31 (Table 1) by using Pfu Turbo Cx Hotstart DNA polymerase (Agilent Technologies) according to manufacturer’s recommendation.

Up- and downstream fragments were made USER compatible by using Pfu Turbo Cx Hotstart DNA polymerase (Agilent Technologies) with oligonucleotide pairs IW28/IW29 and IW32/IW33 (Table 1), respectively.

### USER cloning and plasmids

Up- and downstream fragments of XR and the XR coding sequence containing the point mutation were cloned using simple USER into the USER-compatible vector pSB411, yielding the gene replacement vector pSB411SXR1. This vector was used in PCR as the template for amplifying bipartite fragments used in the transformation of the fungal strain.

### PCR-based bipartite substrates for gene targeting

Bipartite fragments [27] overlapping in the hygromycin selection marker were amplified by PCR from vector pSB411SXR1 using Phusion High-Fidelity DNA polymerase (New England Biolabs) according to manufacturer’s recommendation. *Amplicon 1* was amplified with oligonucleotides IW2 and IW51 (Table 1), and consisted of a 1-kb upstream homologous fragment of the XR gene, the XR coding sequence containing the point mutation, the rp27 ribosomal promoter sequence, and the first two-thirds of the hygromycin gene, having a total length of 3 kb. *Amplicon 2* was amplified with oligonucleotides IW50 and IW7 (Table 1), and contained the last two-thirds of the hygromycin gene, the beta-tubulin terminator sequence, and 1-kb downstream homologous fragment of the XR gene, having a total size of 2 kb.

### Transformation and selection of correct mutant

Protoplasts of *A. carbonarius* ITEM 5010 were prepared from germinated spores after 16 h cultivation in YEPD broth, by digesting the mycelium with 50 ml digestion solution containing 60 mg/ml of cell wall-degrading enzyme mixture (VinoTastePro, Novozymes A/S) for 4 h according to a published protocol [10].

Transformation of the fungus was carried out by adding a total of 2 μg of *amplicon 1* and *amplicon 2* (1:1 molar ratio) in less than 10 μl volume to 100 μl protoplasts, after which the protoplasts were mixed with 1 ml 40 % PEG (Sigma) and resuspended in selective medium. Mitotic stability of the correct mutant (named ‘SXR1’) was achieved by transferring the mutant from selective to non-selective medium, then from non-selective to selective medium. Confirmation of the stable mutant was achieved by performing PCR on the genomic DNA, extracted using the C-Tab Genomic DNA Prep method described elsewhere [40], with oligonucleotides IW17 and IW24 (Table 1), which anneal to the flanking regions of the native XR gene on the genome of the fungus. The mutated XR gene was then sequenced.

Copy number of the insert was determined by the semiquantitative PCR (SQ-PCR) method [8] in the following way. The mutated XR gene (insert, 1,884 bp) was amplified from the genomic DNA of SXR1 with oligonucleotides IW28 and IW31. In parallel, a fragment (genomic fragment) with similar size (2,022 bp) was obtained with forward oligonucleotide IW17, which anneals to upstream of the insert in the original XR region on the genome, and reverse oligo IW31, which anneals to the insert. The PCR reactions were carried out with Run (Taq) DNA polymerase (A&A Biotechnology) in 50-μl reactions. The PCR conditions were similar for the two reactions: initial denaturation at 95 °C for 2 min, followed by 30 cycles of 95 °C for 15 s, 60 °C for 30 s, and 72 °C for 2 min.

Aliquots of 6, 2, and 0.2 % of the total reaction volume of both reactions were analyzed by gel electrophoresis on 1 % agarose gel. Densitometric analysis of the bands was carried out with ImageJ image analysis software [26]. Copy number of the insert was determined from the image analysis results.

### Fermentation setup and conditions

Conidial suspensions of the strains were inoculated into a 50-ml centrifuge tube (Sarstedt) containing 10 ml preculture medium, to give a final concentration of 1 × 10^5^ spores/ml medium, and incubated at 30 °C and 180 rpm for 48 h in a rotary shaker (Ika KS4000i control). Preculture medium contained yeast extract (Sigma), 3.6 g l^−1^ and peptone (Sigma), 10 g l^−1^. Production medium (20 ml) was inoculated with 50 % (v/v) preculture (filtered through sterile Miracloth (Calbiochem) prior to inoculation) and incubated in 100-ml Erlenmeyer flasks at 30 °C and 180 rpm for 7 days in a rotary shaker (Ika KS4000i control). The time of transfer was considered as day 0 and samples were taken on day 7. d-Xylose, citric acid, and xylitol concentrations were analyzed by HPLC on an Aminex 87H column (Biorad). The composition of the production medium was as follows (g l^−1^): d-xylose, 100; NH_4_NO_3_, 2.5; K_2_HPO_4_, 0.1; MgSO_4_·7H_2_O, 1; CaCl_2_·2H_2_O, 0.168; KCl, 0.43; ZnSO_4_·7H_2_O, 4.5 × 10^−3^; FeSO_4_·7H_2_O, 0.75 × 10^−3^ (all from Sigma). The medium was prepared with the aforementioned components and deionized water and autoclaved at 121 °C for 20 min. The fermentation was carried out with triplicate samples for both mutant and wild-type strain. Total dry cell mass was determined by vacuum filtration of the fermentation broth (after day 7 sampling) through preweighed filters (Whatman no. 1) followed by drying at 105 °C to a constant weight.

In this study, three consecutive fermentation experiments were carried out with similar conditions. Dry cell mass was determined after one of the repetitions.

## Results

### Site-directed mutagenesis and gene replacement

Bipartite fragments were used for the transformation in order to achieve higher homologous recombination frequency, for successfully targeting the native XR gene region.

PCR reaction confirmed the successful replacement of the native XR gene with the mutant gene containing the Lys-274 to Arg mutation. After amplification of the mutated gene from the genome, the sequencing results showed the presence of the mutation and excluded any other sequence errors which might have been introduced during the PCR to generate the bipartite fragments.

**Fig. 2:**
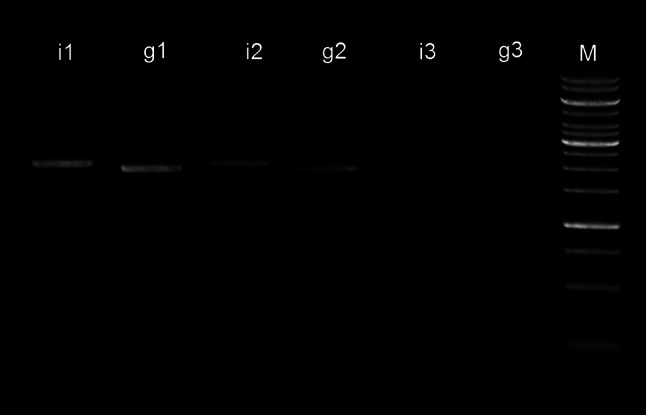
Gel electrophoresis of SQ-PCR reactions. Bands *i1*, *i2*, *i3* represent 6, 2, and 0.2 % of total reaction volume loaded for insert. Bands *g1*, *g2*, and *g3* represent 6, 2 and 0.2 %, respectively, of total reaction volume loaded for genomic fragment.* M* 1-kb DNA ladder

An SQ-PCR approach was used to verify that only a homologous recombination event occurred during the transformation and that no random insertion had happened. The densitometric analysis gave similar relative intensities for band-pairs i1-g1, i2-g2 and i3-g3, after gel electrophoresis (Fig. 2).

### Fermentation with d-xylose

**Fig. 3:**
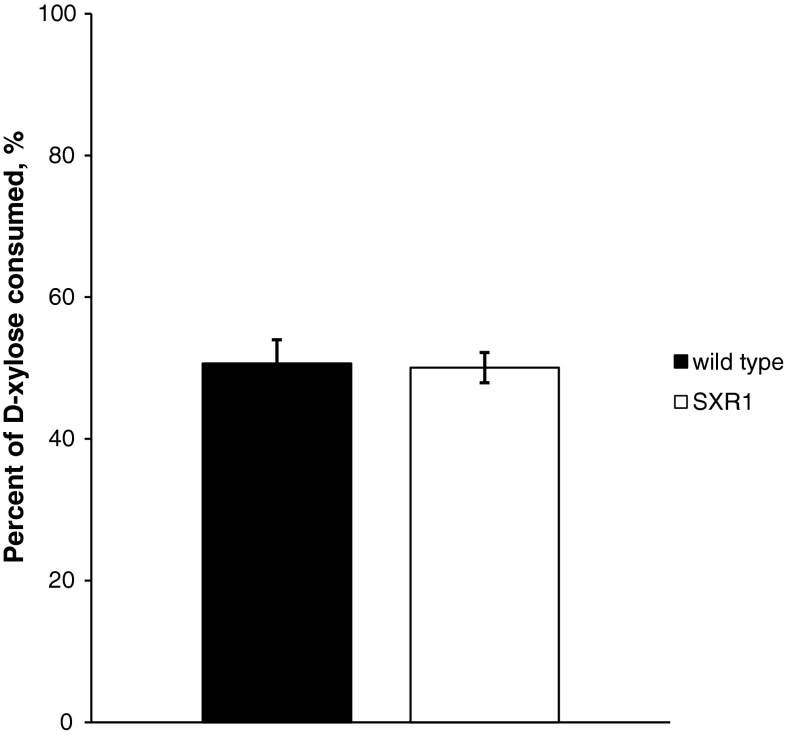
Consumption of the initial d-xylose by the wild-type and mutant strain at day 7 of the fermentation. *Error bars* represent standard deviation between the replicates of the repetitions

Fermentation experiments were carried out in order to detect differences in xylose consumption, xylitol accumulation, and citric acid production between the SXR1 mutant strain and the wild-type strain. The following results are presented as average values of two repetitions of the fermentation involving two replicates of each strain (SXR1 and wild type). No significant differences were noticed between the total sugar consumption after 7 days of fermentation of the wild-type strain and the mutant strain (SXR1) which carried the XR gene with the point mutation. Both strains consumed about 50 % of the initial d-xylose after 7 days of fermentation (Fig. 3). The total dry weight of the mycelium, after 7 days of fermentation, was 438 mg (±14.2 mg) and 403 mg (±16.8 mg) for the wild-type and mutant strain, respectively.

**Fig. 4:**
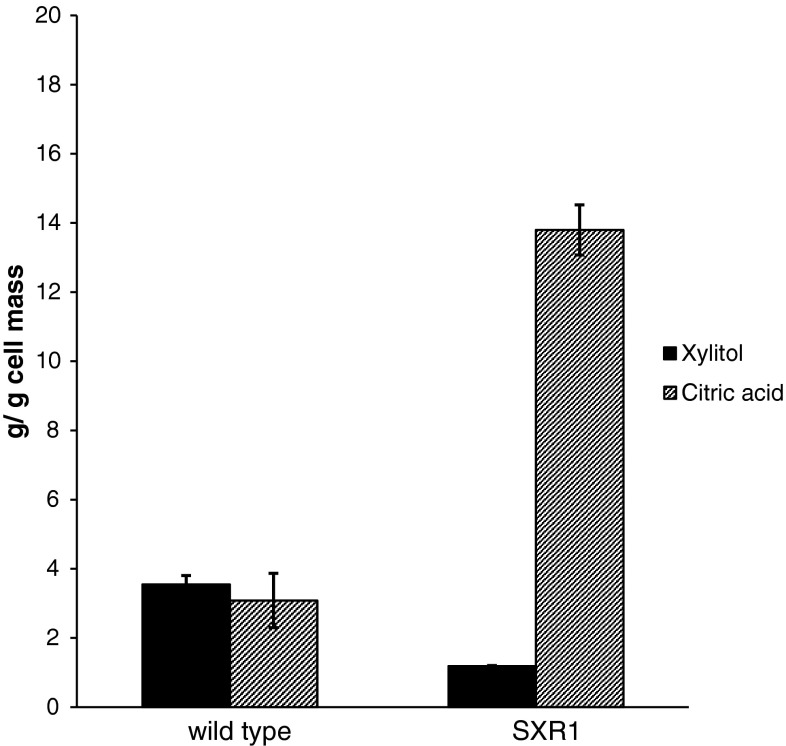
Yield of citric acid and xylitol by the wild-type and mutant strain at day 7 of the fermentation, expressed in grams per gram cell mass. *Error bars* represent standard deviation between the replicates of the repetitions

The successful introduction of the Lys-274 to Arg point mutation in the XR gene of *A. carbonarius* resulted in decreased accumulation of the pathway intermediate, xylitol. The accumulation of xylitol was successfully reduced 2.8-fold compared to the wild-type strain, but it was not completely prevented (Fig. 4). The yield of citric acid was significantly improved (4.5-fold) in the mutant, with a production of 13.8 g citric acid/g cell mass, compared to the yield by the wild-type strain (3.08 g citric acid/g cell formed) (Fig. 4). The results of the fermentations are summarized in Table 2.

**Table 2:** Summary of the fermentation parameters

Strain	Initial d-xylose (g/l)	Final d-xylose (g/l)	Cell mass (g 10^−3^)	Citric acid yield (g/g cell mass)	Xylitol yield (g/g cell mass)
Wild type	93.5	47.34 ± 3.35	438 ± 14.2	3.08 ± 0.79	3.55 ± 0.26
SXR1	93.5	46.78 ± 2.14	403 ± 16.8	13.8 ± 0.72	1.19 ± 0.02

The remaining repetitions of the fermentation experiment resulted in equally improved citric acid yields and reduced xylitol yields (data not shown).

## Discussion

In this study, we have identified a xylose reductase gene in *A. carbonarius* on the basis of sequence homology to the XR gene in *A. niger*. The XR gene contained the conserved Ile-Pro-Lys-Ser motif [22, 30] similar to the NADPH/NADH-dependent XR in *C. tenuis*. We successfully introduced the single point mutation (Lys-274 to Arg) in the xylose reductase (XR) gene in *A. carbonarius*; this mutation was described in *C. tenuis* and was shown to alter the enzyme’s cofactor preference from NADPH towards NADH by causing conformational changes in the coenzyme binding pocket of the enzyme [10]. The point mutation in the *A. carbonarius* mutant resulted in a 2.8-fold reduction of the accumulation of xylitol and a 4.5-fold increase in secretion of citric acid in batch fermentation compared to the wild-type strain. In addition, the fact that all repetitions of the fermentation gave similar results indicates that the performance of the mutant strain is stable and the experiments are reproducible.

When insufficient oxygen is supplied for the cells, the NADH generated by XDH (when xylitol is oxidized to xylulose) accumulates or is regenerated at a slow rate. In this way, no NAD^+^ is available for the XDH and the xylitol (produced by the XR) accumulates and will be excreted [6, 7]. Xylitol accumulation by the wild-type strain implies the suboptimal supply of oxygen for the fungal cells in the current fermentation setup.

Several attempts have been made to improve product yield and prevent xylitol accumulation during ethanol fermentations with yeast. In many reported cases this was successfully achieved by either altering the cofactor preference of XR, so that the redox co-substrates can be recycled in the catalytic steps [31, 32, 39], or by introducing a xylose isomerase [4, 21] which converts d-xylose into d-xylulose in one redox-neutral step. In conclusion, the main factor influencing xylitol accumulation is the cofactor regeneration, when there is insufficient oxygen available for the cells to recycle the NADH produced during the conversion of xylitol to xylulose by XDH.

The fact that the mutant strain shows decreased xylitol levels is assumed to be associated with the capability of the mutated XR to use the NADH generated by the XDH, thus preventing the inhibition of XDH by the high levels of NADH [36] and ensuring the flux of xylose through the PCP as well as a more efficient channeling into consecutive pathways.

In addition, the low levels of xylitol formation in the mutant may be associated with high XR/XDH enzyme activity ratio. Studies show that xylitol accumulation could be totally inhibited in yeast strains with an XR/XDH enzyme activity ratio of 0.06 [38].

In the protoplast-mediated transformation of filamentous fungi, the random integration of foreign DNA is dominant over the site-specific integration [9]. However, the SQ-PCR results indicate that the replacement of the native XR with the mutated gene occurred in strain SXR1 by homologous recombination and the possibility for the introduction of the insert randomly into other regions of the genome can be excluded.

This work shows that enhanced production of citric acid can be achieved using xylose as the sole carbon source by reducing the accumulation of other by-products, such as xylitol. In lignocellulosic biorefineries, yeast is commonly used to ferment the C6 sugars to bioethanol, but cannot utilize the C5 sugars. Thus, there is a need for biocatalysts that can utilize all the sugars in lignocellulose. *A. carbonarius* has this capacity as it is able to co-ferment glucose and xylose (results not shown) and could be an interesting biocatalyst for production of various organic acids or chemical components. If the pretreated biomass is separated into a C5 and C6 stream, the xylose could be used by *A. carbonarius* and will thus reserve the glucose for other biochemical or biofuel fermentations.
